# Leaving trusted paths: Estimating corneal keratometric index in cataract surgery eyes with zero-power implants

**DOI:** 10.1007/s00417-024-06435-8

**Published:** 2024-03-08

**Authors:** Damien Gatinel, Peter C. Hoffmann, David L. Cooke, Alexandre Chingan, Guillaume Debellemanière, Achim Langenbucher, Jascha A. Wendelstein

**Affiliations:** 1 Rothschild Foundation Hospital, Paris, France; 2 Augen-Und Laserklinik, Castrop-Rauxel, Germany; 3 Great Lakes Eye Care, 2848 Niles Road, Saint Joseph, MI 49085 USA; 4 Department of Neurology and Ophthalmology, College of Osteopathic Medicine, Michigan State University, 965 Wilson Rd, East Lansing, MI 48824 USA; 5 Institute of Experimental Ophthalmology, Saarland University, Kirrberger Str. 100/22, 66424 Homburg, Germany; 6 Institut Für Refraktive Und Ophthalmo-Chirurgie (IROC), Stockerstrasse 37, CH-8002 Zurich, Switzerland; 7 Department for Ophthalmology and Optometry, Kepler University Hospital GmbH, Krankenhausstrasse 9, 4020 Linz, Austria; 8 Medical Faculty, Johannes Kepler University Linz, Altenberger Strasse 69, 4040 Linz, Austria

**Keywords:** Keratometry, Keratometric index, Refractive index, Corneal power, Biometry

## Abstract

**Purpose:**

This study aimed to estimate the corneal keratometric index in the eyes of cataract surgery patients who received zero-power intraocular lenses (IOLs).

**Methodology:**

This retrospective study analyzed postoperative equivalent spherical refraction and axial length, mean anterior curvature radius and aqueous humor refractive index to calculate the theoretical corneal keratometric index value (*n*_k_). Data was collected from 2 centers located in France and Germany.

**Results:**

Thirty-six eyes were analyzed. The results revealed a mean corneal keratometric index of 1.329 ± 0.005 for traditional axial length (AL) and 1.331 ± 0.005 for Cooke modified axial length (CMAL). Results ranged from minimum values of 1.318/1.320 to maximum values of 1.340/1.340.

**Conclusion:**

The corneal keratometric index is a crucial parameter for ophthalmic procedures and calculations, particularly for IOL power calculation. Notably, the estimated corneal keratometric index value of 1.329/1.331 in this study is lower than the commonly used 1.3375 index. These findings align with recent research demonstrating that the theoretical corneal keratometric index should be approximately 1.329 using traditional AL and 1.331 using CMAL, based on the ratio between the mean anterior and posterior corneal curvature radii (1.22).


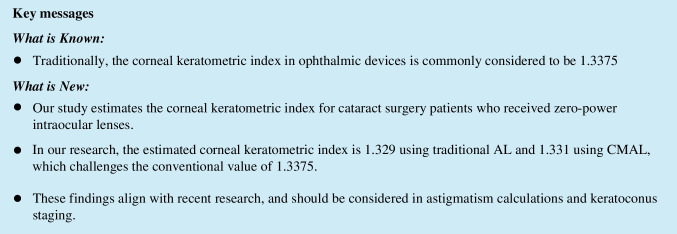


## Introduction

The corneal keratometric index (often denoted as “n” or “n_K_”) plays a pivotal role in various ophthalmic calculations, including intraocular lens (IOL) power selection and corneal refractive surgery. Accurate estimation of this index is essential for achieving optimal visual outcomes. Traditionally, keratometers have used the value *n*_K_ = 1.3375. It is derived from a Gullstrand model cornea, where an *n*_K_ of 1.3375 refers to the back vertex power, an *n*_K_ of 1.332 to the front vertex power, and an *n*_K_ of 1.3315 to the equivalent power. Hence, 7.5 mm (apical anterior corneal radius) coincides with 45 D using the vergence formula. However, this value may be overestimated to approximate the total corneal power.

In this study, we present our findings on estimating *n*_K_ in such cases using a novel approach. We utilized preoperative biometric and postoperative clinical data in high myopic eyes, having received a zero-power IOL. From a paraxial pseudophakic eye model, we could calculate the corneal keratometric index from spherical equivalent refraction, axial length, and mean anterior corneal curvature radius to estimate the corneal keratometric index. Our results challenge the conventional values typically employed in ophthalmic practice and align with recent research suggesting a different theoretical index value based on corneal curvature ratios.

## Patients and methods

### Study design

This retrospective study conformed to ethics codes based on the tenets of the Declaration of Helsinki. Prior ethics approval was obtained (Ärztekammer des Saarlandes, 157/21). We included the eyes of cataract surgery patients with high myopia who had received zero-power implants. An Excel.CSV file (Microsoft Corporation) of postoperative refraction data and preoperative biometry data collected using either the IOLMaster 500 partial coherence interferometry biometry device, or the IOLMaster 700 swept-source optical coherence tomography biometry device of patient exams from two European study centers (Augen-und Laserklinik, Castrop-Rauxel, Germany; Rothschild Foundation Hospital, Paris, France) acquired between 2017 and 2022 were included. Data was anonymized, hence, a backtracing of data was impossible.

Recorded anonymized data included preoperative axial length (AL), flat and steep anterior corneal radii (R1 and R2), central corneal thickness (CCT), anterior chamber depth (ACD), lens thickness (LT), and postoperative spherical equivalent refraction recorded at 6 m using ETDRS charts in center 1 (Rothschild foundation) and at 6 m using Landolt rings in center 2 (Castrop). All data acquisition was executed by experienced staff at the study centers.

### Exclusion of questionable measurement quality

The prerequisite for inclusion was the fulfillment of all instrument-given quality indices (QI) during measurement. The instrument displays QI as “failed,” “warning,” or “successful.” A QI of “failed” or “warning” in any AL or corneal radius measurement led to the exclusion of the whole eye. Duplicate measurements of eyes were omitted.

### Sum-of-Segments Axial Length transformation

Sum-of-Segments AL transformation to Cooke-modified AL (CMAL) was performed in 2 ways:

IOLMaster 700 data was transformed as described in the literature [1, 2].

$$CMAL =1.23853+0.95855\times AL-0.05467\times LT\,+\,0.208.$$

IOLMaster 500 data was transformed using an equation derived from a dataset of 1695 eyes (unpublished data from David L. Cooke):

$$CMAL = 3.57445 +0.61025\times AL +0.014683\times {AL}^{2} -0.0002033806\times {AL}^{3} +0.03847\times ACD+0.208$$

### Calculation of corneal keratometric index:

**Fig. 1:**
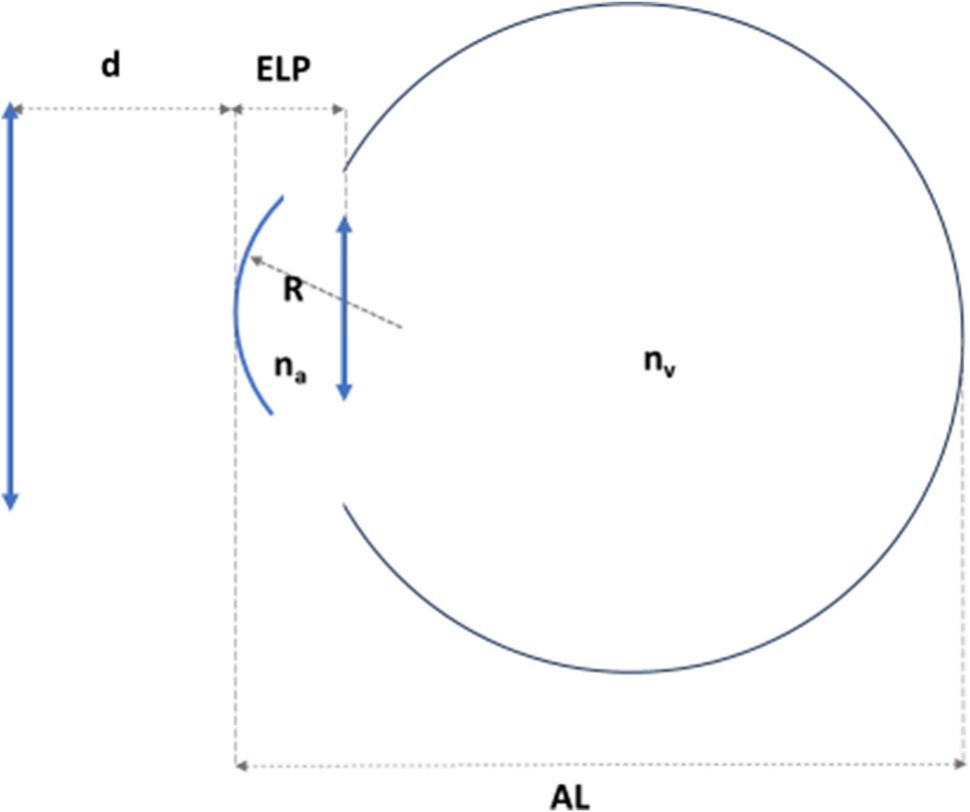
Schematic simplified thin lens pseudophakic eye model. ELP, effective lens position; R, anterior corneal radius; AL, axial length; d, distance from the spectacle plane to the corneal vertex

A simplified thin-lens pseudophakic eye model was built (Fig. 1).

The IOL power of the pseudophakic eye (*P*) is given by:

1 $$P=\frac{{n}_{a}}{AL-ELP}-\frac{{n}_{v}}{\frac{{n}_{a}}{\frac{1}{\frac{1}{SE}+d}\text{ } + \frac{\left({n}_{k}-1\right)}{R}}-ELP}$$

where AL is the axial length, ELP is the effective position of the IOL, SE is the spherical equivalent, R is the anterior corneal radius, *n*_v_ is the refractive index of the vitreous, and *n*_a_ is the refractive index of the aqueous humor. As a simplification, harmonic mean calculation of corneal curvature is used in the processing:

$$R=\frac{2\bullet R1\bullet R2}{(R1+R2)}$$

when *P* = 0, and assuming *n*_a_ = *n*_v_, Eq. 1 can be rearranged as:

2 $$AL= \frac{{n}_{a}}{\frac{1}{\frac{1}{SE}+d}\text{ } + \frac{\left({n}_{k}-1\right)}{R}}$$

Equation 2 can be solved for *n*_k_:

3 $${n}_{k}=1+R\left(\frac{{n}_{a}}{AL}-\frac{SE}{1+d\bullet SE}\right)$$

### Statistical analysis

Data were analyzed using Microsoft Excel (v.16.12, Microsoft Corp) and SPSS software (SPSS V 24.0; IBM, USA). The Shapiro–Wilk test was used to determine the distribution normality of metric variables. Descriptive statistics are provided via tables. Descriptive statistics were used to calculate the mean, standard deviation, median, interquartile range (IQR), 95% confidence interval (CI), minimum, and maximum values of the biometric parameters and corneal keratometric index.

## Results

### Demographic data

A total of 36 eyes from 32 patients met the criteria for inclusion in our analysis dataset.

Male/female ratio was 0.44, OD/OS ratio was 0.88.

Zero power IOL models implanted were 17 Asphina (Carl Zeiss, Jena, Germany), 12 MA60MA (Alcon, Geneva, Switzerland), 7 MX60 (Bausch and Lomb, Laval, Canada).

Table 1 shows the descriptive data of the biometric measures of all eyes in terms of AL, CCT, ACD, LT, and the radii of curvature for the corneal front surface and the computed keratometric index values (*n*_K_).

**Table 1:** Preoperative biometry derived data, refraction data, and computed keratometric indices calculated with traditional axial length (*n*_K(AL)_) and sum-of-segments axial length (*n*_K(CMAL)_)

	AL	CMAL	ACD	LT	CCT	Ra	SE (D)	Computed *n*_K(AL)_	Computed *n*_K(CMAL)_
MEAN	32.316	32.189	3.446	4.364	0.547	7.557	− 2.108	1.329	1.331
SD	1.449	1.395	0.488	0.377	0.043	0.202	1.752	0.005	0.005
Median	32.316	32.215	3.450	4.347	0.540	7.590	− 2.000	1.329	1.331
IQR	1.640	1.603	0.285	0.173	0.046	0.303	2.625	0.006	0.006
MIN	28.950	28.977	2.440	3.610	0.496	7.110	− 5.750	1.318	1.320
MAX	36.170	35.842	4.470	5.400	0.656	7.880	0.500	1.340	1.340
5% quantile	29.825	29.802	2.472	3.636	0.496	7.224	− 5.531	1.319	1.320
95% quantile	35.400	35.086	4.446	5.153	0.635	7.867	0.391	1.337	1.339
Lower 95% CI	31.843	31.733	3.287	4.241	0.533	7.491	− 2.680	1.328	1.329
Upper 95% CI	32.790	32.645	3.606	4.487	0.561	7.623	− 1.535	1.331	1.332

*AL* axial length in mm, *CMAL* Cooke-modified axial length in mm, *ACD* anterior chamber depth in mm, *LT* lens thickness in mm, *CCT* central corneal thickness in mm, *Ra* mean anterior corneal curvature in mm, *SE* postoperative spherical equivalent refraction in diopters

Data were normally distributed. Our analysis revealed a mean *n*_K(AL)_ of 1.329 ± 0.005 and a mean *n*_K(CMAL)_ of 1.331 ± 0.005, with a lower 95% CI of 1.328/1.329 and an upper 95% CI of 1.331/1.332.

**Fig. 2:**
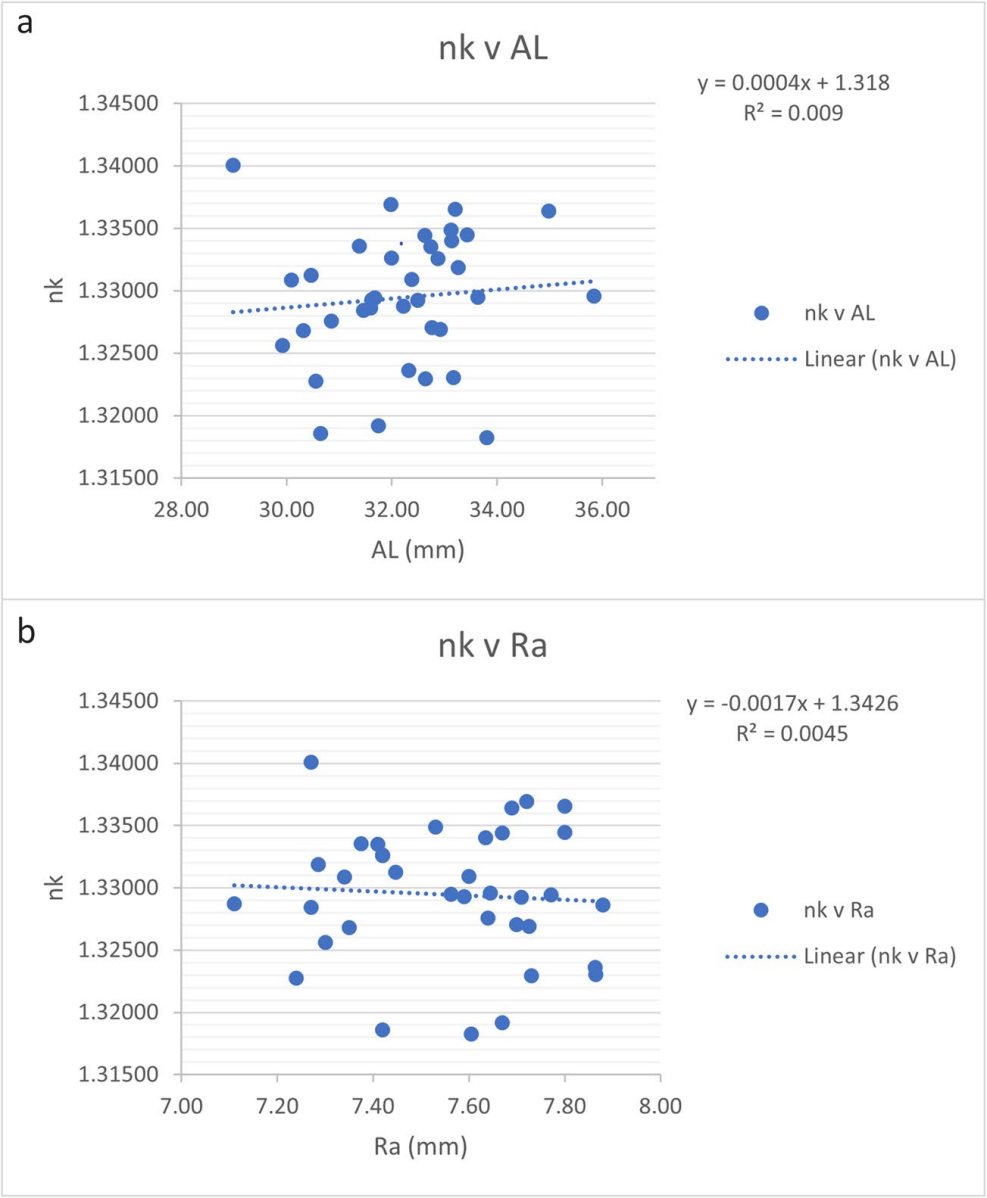
**a**, **b** Trend errors of the keratometric index *n*_K(AL)_ calculated with traditional axial length when plotted against axial length in mm and the harmonic mean of the anterior corneal curvature in mm

**Fig. 3:**
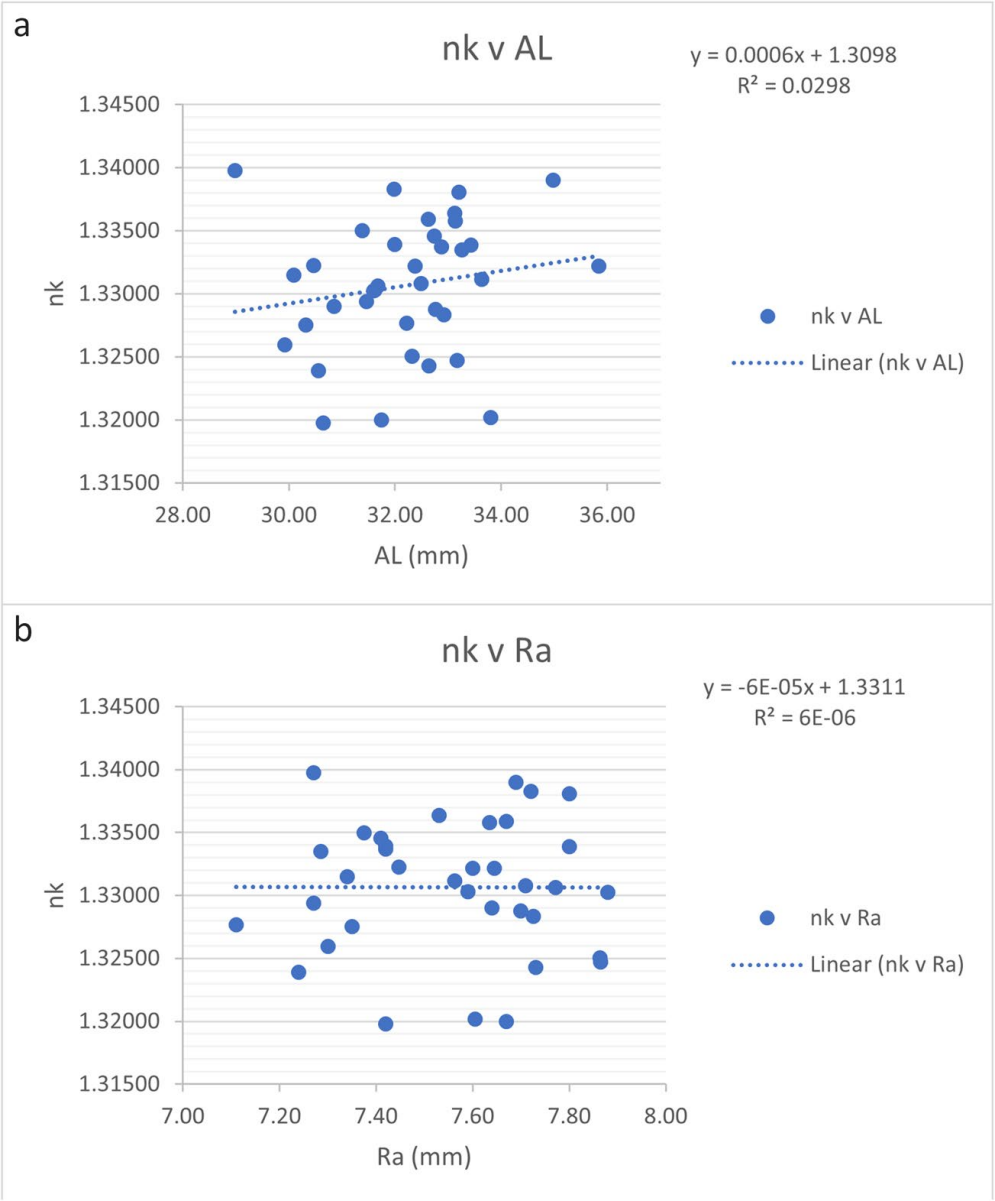
**a, b** Trend errors of the keratometric index *n*_K(CMAL)_ calculated with a sum-of-segments axial length when plotted against axial length (AL) in mm and the harmonic mean of the anterior corneal curvature (Ra) in mm

Figure 2a depicts dependencies of *n*_K(AL)_ and AL. Figure 2b depicts trend errors for *n*_K_ against R. There were almost no dependencies (*R*^2^ = 0.009 for AL and *R*^2^ = 0.0045 for Ra) on either parameter. Figure 3a depicts dependencies of *n*_K(CMAL)_ and CMAL. Figure 3b depicts trend errors for *n*_K(CMAL)_ against R. There were almost no dependencies (*R*^2^ = 0.0298 for CMAL and *R*^2^ < 0.0001 for R) on either parameter.

## Discussion

Historically, biometers have measured the anterior corneal power and empirically factored in the power contribution from the posterior cornea using a hypothetical keratometric index. This corneal keratometric index is a crucial parameter in ophthalmic practice, influencing surgical outcomes and optical calculations. Unfortunately, measuring corneal power is not feasible when the cornea is in its natural position. When interpreting corneal shape for conversion to corneal power, we require a supporting optical model that provides the refractive indices of corneal tissue and aqueous humor. Assumptions are often made, typically derived from a schematic model eye. Traditionally, it has been assumed to have a standard *n*_K_ value of 1.3375, which is also used to generate keratometric axial and tangential corneal power maps, astigmatism vectors and indices for keratoconus detection/staging. Usually, biometric and tomographic devices work with corneal input data based on *n*_K_ 1.3375, and third and fourth-generation IOL power formulas either directly use this value or internally transform corneal power to specific *n*_K_ values (HofferQ uses 1.3375, Haigis 1.3315, Holladay 1.333, and SRK/T 1.333) [3–6]. New generation formulae also typically base input data on *n*_K_ 1.3375; however, in undisclosed formulae, it is unknown which internal *n*_K_ corneal data is transformed to. Our study, in alignment with recent research, demonstrates that, on average, these values tend to overestimate the total corneal power.

In a Gullstrand model cornea, 1.3375 refers to back vertex power, 1.332 to front vertex power, and 1.3315 to equivalent power. Even if there is not a large gap between principal plane and front apex plane, this gap depends on corneal thickness and the Coddington factor (who indicates how much the curvature should deviate from a perfect spherical shape to minimize spherical aberration) and cannot be evaluated by a standard keratometer. In principle, all segment distances in biometry are measured from the front apex plane; therefore, it makes sense to use the front apex plane as a reference plane to base *n*_K_ on. Indeed, the *n*_K(CMAL)_ we found in this study, using a thin lens cornea and a sum-of-segments approach for AL measurements, is a bit lower than the *n*_K_ of 1.332 referenced to the front apex plane in a Gullstrand model cornea. Additionally, not only *n*_K(CMAL)_ but also *n*_K(AL)_ based on traditional AL, reveals that *n*_K_ in our study population of mainly highly myopic eyes with zero power IOLs was considerably lower than 1.3375. This is consistent with our previous publications on this topic [7–9]. In the study at hand, we observed a mean *n*_K(AL)_ value of 1.329 ± 0.005. This observation aligns with our previous research, which provided an equation to compute the optimal *n*_K_ value to match the total corneal power for a given corneal mean radii anterior to posterior ratio. It suggested that the theoretical corneal keratometric index should be closer to 1.329, based on the ratio between the mean anterior and posterior corneal curvature radii (1.22) [7].

In our prior study involving a sample size of 99 eyes and utilizing three eye models (including thin lens and thick lens models), we back-calculated the keratometer index based on a vergence formula. We obtained values of 1.331 ± 0.003, 1.331 ± 0.003, and 1.332 ± 0.003, based on the subjective refraction, accounting for the refractive measurement distance. This fits the *n*_K(CMAL)_ observed in the current dataset. Importantly, in neither of the 2 studies were trend errors observed with axial length or corneal radius (Figs. 2a, b and 3a, b), suggesting the credibility of using an *n*_K_ of below 1.332 for vergence calculation [8]. In another larger study, we developed a raytracing-based strategy for calculating corneal power from anterior segment optical coherence tomography data and extracted individual keratometer indices. Again, this approach yielded an average *n*_K_ of 1.332 ± 0.002, with a median of 1.332 and a range from 1.323 to 1.339 [9]. Furthermore, we were able to indicate that the performance of some classic IOL calculation formulae could in general be improved by variation of *n*_K_, as opposed to using the formula specific index [10]. We were able to reduce the trend error of PE for the corneal radius with variation of the formula constants and *n*_K_. More realistic *n*_K_ values could therefore help to reduce trend errors in formulae but require new formula constants and/or, depending on the ELP algorithm, the use of a “Double-K” concept (or a recalibration of the ELP algorithm) [10, 11].

The even lower *n*_K(AL)_ observed in our current dataset may be explained by the use of eyes with zero-power IOLs, ruling out any influence from wrong assumptions of the ELP. Yet, the corneal radii of such highly myopic eyes may fall outside the normal range, which could introduce some measurement errors when recording radii with a keratometer. Furthermore, the use of sum-of-segments AL in our dataset may be more important than in other datasets containing larger numbers of eyes with normal ranges of AL. It is well known that traditional optical biometry AL ignores the segment lengths and calculates AL based on the locations of the corneal front apex and the retinal pigment epithelium (RPE) using mathematical conversions to the inner limiting membrane (ILM) [6]. In turn, the transformation seems to overestimate AL in eyes with larger AL, which leads to hyperopic prediction errors in myopic eyes with classical IOL formulae [12]. Ideally, biometers would stop converting AL to the ILM and report the measured AL from the corneal front apex to RPE, which we tried to account for using CMAL in our study [1, 2]. We specifically moved the CMAL to the RPE by adding 207.8 µm to it, as already performed in a previous publication that approximated CMAL and the sum-of-segments AL [2]. Our *n*_K(CMAL)_ may therefore more truthfully represent corneal power than *n*_K(AL)_. We did not find dependencies on AL/CMAL or corneal curvature for our data, showing that our lower K Index seems to be credible, at least in a population of highly myopic people, considering that there was not much variation of AL in our dataset due to the use of zero-power IOLs. It is rather interesting that *n*_K(AL)_ was in line with the Liou Brennan model, whereas *n*_K(CMAL)_ was in between the Liou Brennan model and the Gullstrand model referenced to the front vertex power. Differences in *n*_K(CMAL)_ and *n*_K(AL)_ should become less pronounced in eyes with shorter AL, as CMAL starts to subtract from AL at around 27 mm and larger. This assumption may not be fully true as the CMAL correction is partly constructed in accordance to the proportion between the crystalline lens and vitreous. Ideally, the post-operative AL with a zero-power IOL should be measured to rely less on assumptions about the refractive index of the lens. Unfortunately, postoperative AL measurements were not available for our study dataset. The variation in *n*_K_ within our dataset was notable but not excessive; with a standard deviation (SD) of 0.005 it was a bit higher than in our two predecessing studies where SD was 0.002 and 0.003 [8, 9]. We also have to consider that myopic corneas may have a different spherical aberration than emmetropic or hyperopic corneas [13]. Notably, in previous studies, we found that front surface asphericity had an impact on the keratometer index [9].

Most IOL power calculation formulas are primarily established for use with a keratometric index comprised between 1.3315 and 1.3375, and it is also important to recognize that many biometric values are approximated to older measurements, e.g., AL is transformed to ultrasound measurement which only measures to ILM; other examples are ACD and LT. Among other reasons, this is mainly due to the wish to keep established IOL constants available on compository websites the same and to be able to receive approval from the U.S. Food and Drug Administration for newer biometric devices. The established constants attempt to compensate for these approximations and model errors of the formula. For toric IOLs, especially in cases of higher astigmatism, the discrepancy between an *n*_K_ of 1.3375 and a more realistic *n*_K_ becomes more pronounced. Unlike other calculations for toric IOLs, toric magnitude calculation does not require formula constants, making a case for the use of a more realistic *n*_K_ in astigmatism display, as already described by Holladay in 2001 [14]. As for Keratoconus progression criteria, a K-max value of 60 D displayed with a keratometric index of 1.3375 amounts to a radius of 5.625 mm and if reconverted with our new *n*_K(AL)_ of 1.329 to only 58.49 D and with *n*_K(CMAL)_ to 58.67 D. Care has to be taken when interpreting such values that all display modes use the same keratometric index, or the minimum radius may be taken as more independent marker for progression.

One notable advantage of our study is the use of eyes implanted with IOLs with a power of zero D, which provided valuable insights and minimizes any confounding effects of the ELP that may have been present in earlier studies on this topic. It is of note though, that while zero-power IOLs do not have any focusing effect, they can widen the light beam, as explained by Haigis for the case of the MA60MA IOL: He estimated the effect to 0.26 D the hyperopic shift induced by the zero-power MA60MA for an eye with an AL of 31.56 mm [15]. Another limitation is the lack of knowledge regarding the optical path length that allows for an even more precise estimation of the keratometric index.

Our results emphasize the importance of considering patient-specific factors in keratometric index calculations. This study carries significant implications for intraocular lens power calculations and corneal refractive surgery planning, highlighting the need for customized approaches to achieve optimal visual outcomes in cataract surgery patients. Further research is warranted to validate and refine these findings.
